# Supplementary material for: Targeting of nanoparticles to the cerebral vasculature after traumatic brain injury
Source: PLoS One. 2024 Jun 10;19(6):e0297451. doi: 10.1371/journal.pone.0297451 (PMC11164327; doi:10.1371/journal.pone.0297451)
Supplement: S1 Fig — N = 6, mean±SEM. (DOCX) [file pone.0297451.s001.docx]

**Figure S1. Recovered cells in TBI brain. N**=6**, mean**±SEM
