# Supplementary material for: Targeting of nanoparticles to the cerebral vasculature after traumatic brain injury
Source: PLoS One. 2024 Jun 10;19(6):e0297451. doi: 10.1371/journal.pone.0297451 (PMC11164327; doi:10.1371/journal.pone.0297451)
Supplement: S1 Table — N≥3, mean±SEM. (DOCX) [file pone.0297451.s002.docx]

**Targeting of nanoparticles to the cerebral vasculature after traumatic brain injury**

**Authors**

Serena Omo-Lamai^1¶^, Jia Nong^2¶^, Krupa Savalia^3¶^, Brian J. Kelley^4¶^, Jichuan Wu^5^, Sahily Esteves-Reyes^6^, Liam S. Chase^5^, Vladimir R. Muzykantov^2^, Oscar A. Marcos-Contreras^2^, Jean-Pierre Dollé^4^, Douglas H. Smith^4^*, Jacob S. Brenner^2,5^*

* contributed equally

** to whom correspondence should be addressed

**Table S1. Biodistribution of mAb in sham vs TBI mouse. N**>**3, mean**±SEM

|  | % of injected dose per gram of tissue | | | | Average tissue weight  (gram) |
| --- | --- | --- | --- | --- | --- |
|  | Sham | | TBI | |  |
|  | IgG | VCAM | IgG | VCAM |  |
| Blood | 45.26±3.75 | 8.71±0.56 | 56.11±1.15 | 12.97±0.53 | 1.6 |
| Lung | 1.32±0.34 | 12.50±1.26 | 2.32±1.03 | 10.38±0.48 | 0.15 |
| Heart | 4.10±1.12 | 9.64±0.40 | 3.83±0.72 | 8.63±1.12 | 0.1 |
| Liver | 8.09±0.49 | 13.78±0.28 | 10.11±1.54 | 14.01±0.55 | 1.1 |
| Kidney | 7.81±2.21 | 13.54±0.65 | 15.21±0.23 | 17.08±1.47 | 0.3 |
| Spleen | 12.41±1.43 | 141.25±1.49 | 8.30±0.38 | 184.31±17.28 | 0.08 |
| Brain | 0.36±0.01 | 2.06±0.25 | 0.67±0.05 | 1.68±0.10 | 0.45 |
