# Supplementary material for: Targeting of nanoparticles to the cerebral vasculature after traumatic brain injury
Source: PLoS One. 2024 Jun 10;19(6):e0297451. doi: 10.1371/journal.pone.0297451 (PMC11164327; doi:10.1371/journal.pone.0297451)
Supplement: S2 Table — N≥3, mean±SEM. (DOCX) [file pone.0297451.s003.docx]

**Table S2. Biodistribution of liposomes in sham vs TBI mouse. N**>**3, mean**±SEM

|  | % of injected dose per gram of tissue | | | | Average tissue weight  (gram) |
| --- | --- | --- | --- | --- | --- |
|  | Sham | | TBI | |  |
|  | IgG | VCAM | IgG | VCAM |  |
| Blood | 28.70±3.18 | 26.0±4.75 | 25.15±7.23 | 9.38±0.48 | 1.6 |
| Lung | 3.63±0.18 | 7.65±0.35 | 3.07±0.52 | 12.79±0.24 | 0.15 |
| Heart | 1.48±0.36 | 4.50±0.75 | 0.63±0.17 | 7.79±0.86 | 0.1 |
| Liver | 30.60±2.53 | 23.62±0.13 | 31.92±4.40 | 23.20±0.71 | 1.1 |
| Kidney | 3.88±0.92 | 12.07±1.02 | 3.66±0.77 | 15.49±0.65 | 0.3 |
| Spleen | 36.14±4.56 | 73.67±2.67 | 50.23±16.67 | 114.96±7.75 | 0.08 |
| Brain | 0.38±0.19 | 0.87±0.15 | 0.18±0.05 | 1.83±0.16 | 0.45 |
