# Supplementary material for: Targeting of nanoparticles to the cerebral vasculature after traumatic brain injury
Source: PLoS One. 2024 Jun 10;19(6):e0297451. doi: 10.1371/journal.pone.0297451 (PMC11164327; doi:10.1371/journal.pone.0297451)
Supplement: S3 Table — N = 6, mean±SEM. (DOCX) [file pone.0297451.s004.docx]

**Table S3. Recovered Cells in TBI brain. N**=6**, mean**±SEM

|  | Mean | SEM |
| --- | --- | --- |
| Endothelial Cells | 9.43 | 1.08 |
| Leukocytes | 36.35 | 3.72 |
| Microglia (CD45mid) | 12.37 | 1.25 |
| Double Negative | 40.02 | 5.13 |
