# Supplementary material for: Targeting of nanoparticles to the cerebral vasculature after traumatic brain injury
Source: PLoS One. 2024 Jun 10;19(6):e0297451. doi: 10.1371/journal.pone.0297451 (PMC11164327; doi:10.1371/journal.pone.0297451)
Supplement: Attachment — Submitted filename: Response to Reviewers.docx [file pone.0297451.s005.docx]

We thank all four reviewers for their constructive feedback and suggestions for our manuscript throughout the entire editorial process. In order to address these comments as thoroughly as possible, we have performed additional experiments and have reanalyzed the existing data. Our point-by-point response to the specific questions raised by the reviewers can be found below (in blue). All changes to the manuscript are denoted with blue text.

Reviewer #1: This manuscript reports on targeting attempts to enable uptake of anti VCAM-antibody targeted liposomes in an experimental model of traumatic brain injury (TBI). This manuscript provides novel observations but revisions are needed.

1) It is said that the blood-brain barrier (BBB) is permeable for years in TBI. Relevant citations are provided, but it would improve this statement if the authors could describe some more on the evidence for this disruption (increases in paracellular or transcellular, persistent inflammation, quantitative measures, compensatory changes in TJ-protein expression patterns etc)

We thank the reviewer for raising the question of BBB permeability in TBI. TBI induced BBB dysfunction has been attributed to an increase in paracellular transport through the loss of tight junction proteins and an increase in larger molecules and proteins through transcytosis. In order to demonstrate such disruption, we compared the accumulation of untargeted IgG antibody in naïve vs. TBI brain. IgG has a half-life of 6-8 days in the blood stream in mice, and our data shows that only a tiny fraction enters the brain in naïve mice. However, when there is capillary leakage, IgG will extravasate into the brain together with other plasma protein. The result below shows ~8-fold increase of IgG in the TBI brain, when compared to the naïve brain, suggesting the disruption of BBB. Dashed lines representing the naïve level for IgG and anti-VCAM-1 antibody have been added into Fig 1D and 1E. The following text has been added in the Results section, with additional references in the Introduction section.

It is worth noting that the IgG accumulation in the TBI brain is ~8 fold higher than that of naïve brain (black dashed line). Notably, IgG circulates well and accumulates minimally in the healthy brain. These results suggested that TBI induces capillary leakage, though at this point we cannot say if IgG extravasation into the TBI brain is due to transport that is transcellular, paracellular, or via frank hemorrhage. Additionally, the extravasated IgG in TBI brain contributed to the lower fold-improvements of VCAM-1 antibody vs. IgG control in TBI vs. sham brain.

2) The VCAM targeting approach is not all new. Adequate citation is needed (e.g. PMID: 24389338)

We thank the reviewer for the suggestion and have included a few more citations regarding VCAM-1-targeting strategies.

3) VCAM: Change to VCAM-1 (There are more VCAM's)

Thank you for pointing this out. We have changed the manuscript to use the term VCAM-1 throughout the text.

4) The abstract does not read that well: It says testing in TBI increases uptake of VCAM-1-liposomes compared to non-targeted 10-fold. And 2-fold compared to sham (non-TBI ?), but then it says " VCAM antibodies did not demonstrate a similar enhancement in brain uptake following TBI". How does that relate?

We thank the reviewer for pointing out the unclarity and have updated the abstract.

Traumatic brain injury has faced numerous challenges in drug development, primarily due to the difficulty of effectively delivering drugs to the brain. However, there is a potential solution in targeted drug delivery methods involving antibody-drug conjugates or nanocarriers conjugated with targeting antibodies. Following a TBI, the blood-brain barrier (BBB) becomes permeable, which can last for years and allow the leakage of harmful plasma proteins. Consequently, an appealing approach for TBI treatment involves using drug delivery systems that utilize targeting antibodies and nanocarriers to help restore BBB integrity. In our investigation of this strategy, we examined the efficacy of free antibodies and nanocarriers targeting a specific endothelial surface marker called vascular cell adhesion molecule-1 (VCAM-1), which is known to be upregulated during inflammation. In a mouse model of TBI utilizing central fluid percussion injury, free VCAM-1 antibody did not demonstrate superior targeting when comparing sham vs. TBI brain. However, the administration of VCAM-1-targeted nanocarriers (liposomes) exhibited a 10-fold higher targeting specificity in TBI brain than in sham control. Flow cytometry and confocal microscopy analysis confirmed that VCAM-1 liposomes were primarily taken up by brain endothelial cells post-TBI. Consequently, VCAM-1 liposomes represent a promising platform for the targeted delivery of therapeutics to the brain following traumatic brain injury.

5) It is well-known that VCAM-1 liposomes target the brain endothelium, and this may have therapeutic potential, e.g. to enable drug delivery specifically to enhance the endothelium to repair after TBI. A question would be how much is getting further into the brain since the BBB is said to be open for long and whether is could be transformed into something therapeutically useful even when attempted targeted to the brain endothelium (VCAM-1 is probably also expressed by astrocytes). The morphological data of Fig 2 does suggest the liposomes moving further into the brain; please this fact in relation to the proclaimed higher transport after TBI.

Great point. We agree with the reviewer that VCAM-1 has been reported to be expressed on many cell types, including astrocytes. However, with our method (30-minutes post-IV injection), it is unlikely that the liposomes will reach astrocytes due to the distance between the endothelium and astrocytes, and basal lamina/pericytes in between. In this paper, we primarily focused on the endothelial targeting because we believe that repairing the leaky BBB is the most immediate task to prevent the brain damage from leakage of toxic plasma across the BBB. Our flow cytometry data has demonstrated that the majority of the liposomes are associated with endothelial cells. However, since it is a very important topic of nanocarrier pharmacokinetics, in future studies, we will address the location within other cell types with an extended time courses. The following text has been added into the Discussion section.

In addition, our flow cytometry data and histology images have demonstrated that the majority of the VCAM-1-targeted liposomes are associated with endothelial cells with 30 minutes post-IV injection. These results align with our primary focus on endothelial targeting because we believe that repairing the leaky BBB is the most immediate task to prevent the brain damage from leakage of toxic plasma across the BBB. However, since VCAM-1 has been reported to be expressed on many other cell types, including astrocytes, future studies of nanocarrier pharmacokinetics and cellular distribution will be performed with an extended time courses.

Reviewer #2: In this manuscript, authors suggest that VCAM liposomes efficiently target the brain in TBI mice, especially endothelial cells. Their results imply that VCAM liposome may be a novel strategy for drug delivery to the brain and targeting the BBB. However, some points need to be improved for publication in the PLOS ONE.

1) According to Fig.2B, VCAM liposomes are predominantly distributed in the spleen. The brain content of VCAM liposomes may be fairly low. How percentage of VCAM liposomes is achieved in the brain by IV administration? Data is needed to be shown.

We thank the reviewer for the astute comment. In targeted drug delivery of TBI, the goal is to increase the concentration of drugs reaching the BBB endothelium. While it would be ideal for the nanocarriers to only go to the BBB, in practice, all nanocarriers, including the ones in this manuscript, also deliver to off-target organs, especially the liver and spleen. However, as long as the cargo drugs are not toxic to these remote organs, great benefit can be derived from the increase concentration of the cargo drug at the BBB, compared to free drug delivery. Additionally, for nucleic acid cargos, such as mRNAs delivered by lipid nanoparticles, which we are now developing for TBI, the cargo mRNAs could achieve literally zero expression in the BBB without the type of targeting we use here. Thus, the targeted nanoparticles in this manuscript have as their goal to massively augment the delivery of cargo drugs to the BBB endothelium compared to delivery without a nanocarrier. The issue of concomitant delivery to other organs, such as spleen, are important, and must be considered when choosing cargo drugs. We have now added the following text to explain this more fully.

Additionally, since the reviewer mentioned the spleen, we will specifically discuss that organ. The spleen has a large number of VCAM-1 epitopes, but as explained below, on a per-gram basis, the brain in TBI mice has more VCAM-1-specific uptake than does the spleen. We arrive at that conclusion by normalizing to VCAM-1 nanoparticle uptake in each organ by the concentration in the blood, thus eliminating the blood pool’s effects, since spleen has a very large volume of blood compared to parenchyma. We additionally normalize by the organ uptake of a non-specific control, IgG. This immunospecificity index (se calculation equation below) show that spleen did not demonstrate significant specificity for VCAM-1 targeting compared to the brain. The calculation and the following text have been included in the Results section.

Since spleen is the major reservoir of VCAM-1 (shown in **Fig 1F**) and clearance organ of nanocarriers, we wanted to evaluate the immunospecificity (IS) of VCAM-1 targeted liposomes in spleen and brain, by accounting for the effect of blood driven delivery and non-specific delivery of IgG control. Using the IS index calculation: (%ID/g VCAM-1-organ/%ID/g VCAM-1-blood)/( (%ID/g IgG-organ/%ID/g IgG-blood), we found that in sham animals, the IS index of brain and spleen are similar (2.539±0.431 vs. 2.251±0.082, *p*=0.055). However, in TBI animals, the IS index of brain in significantly higher than spleen (14.805±1.273 vs. 9.735±0.657, *p*=0.024). It suggests that VCAM-1-targeted liposomes have specific targeting to the TBI-injured brain.

The brain uptake of VCAM-1 liposomes is ~0.8 % of injected dose (%ID). This is 133-fold and 23-fold higher than untargeted IgG liposomes (0.006 %ID) and transferrin receptor targeted liposomes (0.034 %ID) from published data. The numbers are added in Table S1 and S2.

2) I wonder why VCAM liposomes had a higher brain uptake than IgG liposomes in the TBI brain. Please describe in the discussion.

We thank the reviewer for the comment and we have added the following text in the Discussion section.

Acute brain injuries of various ethiologies display the common pathology of neurovascular inflammation. Upon inflammation, endothelial cells along the BBB are activated and overly express cellular adhesion molecules, including VCAM-1. In contrast to IgG, which is constantly recycled back to the plasma via neonatal Fc receptor after cell uptake, IV administration of VCAM-1 antibody binds endothelium once entering the circulation. The high accessibility of the BBB endothelium (surface area of ~20 m^2^) and upregulated expression of VCAM-1 on the BBB endothelium upon neurovascular inflammation in TBI make VCAM-1 a promising target for drug delivery to the brain.

3) In the Text, Fig1B, C, D, E, and F were not correctly applied. Please checked carefully.

It has been corrected in the main text.

Reviewer #3: The article by J. Nong et al aims at using a targeted drug delivery approach towards treatment of traumatic brain injury (TBI), which is a quite relevant topic to address. Thus, the authors proposed the use of liposomes decorated with antibodies specific for VCAM (vascular cell adhesion molecule), which is a specific endothelial surface marker, and compared their targeting ability with that of the free antibodies in a mouse model of TBI utilizing central fluid percussion. The main conclusion drawn is that VCAM-liposomes are a promising delivery system for the targeted delivery of therapeutics to the brain following traumatic brain injury. Indeed, VCAM-targeted liposomes have shown a 10-fold higher accumulation in the brain compared to untargeted liposomes, and brain uptake of VCAM-liposomes increased 2-fold in comparison to sham mice. Interestingly, flow cytometry and confocal microscopy analysis demonstrated that VCAM liposomes were primarily taken up by brain endothelial cells post-TBI.
The study presented herein is quite relevant considering the importance of crossing BBB for treating brain diseases. The work is sound and the conclusions are in accordance with the main results obtained. Therefore, the article can be accepted in PLOS after minor revision:

Pages 9/10 - Antibody modification. It seems that there is a missing reference: “ …For biodistribution studies, monoclonal antibodies were radiolabeled with Na125I using Pierce Iodogen radiolabeling method (add reference?). Briefly, tubes…”;

The reference has been added.

Page 12 - Please replace (Fig 1E) by (Fig 1F);

It has been replaced in the main text.

Page 13 – Discussion. The authors state that “Finally, we showed that the biodistribution of VCAM-free antibodies does not reliably predict that of VCAM-nanocarriers, as only VCAM conjugated-nanocarriers, but not VCAM antibody, display augmented brain uptake upon TBI.” This issue should be discussed into more detail. Is there a tentative reason that explain this behavior? Are there any experimental results of Nong´s Group or literature data that could explain the difference between of VCAM-nanocarriers and free antibodies?

We appreciate the comment and the following text has been added the Discussion section.

In retrospect, it might be surprising that the mAb and nanocarrier versions of anti-VCAM-1 produce different pharmacokinetics, as the mAb and nanocarriers are different in size, valency, and accessibility to targets. For example, in the context of the disrupted BBB after TBI, IgG at the size of 4~15 nm, should be able to extravasate across disrupted BBB and accumulate in the brain parenchyma, while the nanocarrier with the size of ~145 nm, cannot directly go through the disrupted BBB. Additionally, nanocarriers are easily recognized by phagocytes once entering the blood stream and accumulate in the liver and spleen. Our lab has extensively studied the comparison of antibody vs. nanocarriers in various animal models of acute injuries, and have previously found other pathologies (e.g., acute lung injury) that dramatically change the pharmacokinetics profiles of antibodies and nanocarriers.

Reviewer #4: The manuscript by Omo-Lamai et al focuses on a vascular cell adhesion molecule (VCAM) -targeting liposome to target inflamed endothelium after TBI. Using a mouse model of TBI, this study shows that intravenous administration of VCAM-targeted liposomes exhibits higher accumulation accumulation in TBI mice, as compared to healthy mice. Flow cytometry and confocal microscopy analysis confirmed that VCAM liposomes were primarily taken up by brain endothelial cells post-TBI. Use of VACM-targeted nanoparticles for different brain applications has been demonstrated previously in multiple papers. Therefore, novelty of this work is limited. The claims are not justified by the data. For example, the authors mention in the abstract that
“intravenous administration of VCAM-targeted nanocarriers (liposomes) exhibited a 10-fold higher accumulation in the brain compared to untargeted liposomes.” But there is no data showing accumulation/uptake of untargeted liposomes. The work is also extremely limited with just looking at brain accumulation, and data has been duplicated in figures. Please see below for a few more specific comments:

We thank the reviewer for the thorough comments. We have clarified in the text that we used IgG-conjugated liposomes as the untargeted control for VCAM-1-targeted liposomes. In contrast to many studies which are only focused on the organ of interest, isotope tracing allows us to investigate the biodistribution of blood and major organs without tissue homogenization, as shown in Figure 1F and 2B. With the information of whole body biodistribution, we were able to determine the targeting specificity of VCAM-1 in sham vs TBI-challenged animals. We have included the following text in the Results section.

Since spleen is the major reservoir of VCAM-1 (shown in **Fig 1F**) and clearance organ of nanocarriers, we wanted to evaluate the immunospecificity (IS) of VCAM-1 targeted liposomes in spleen and brain, by accounting for the effect of blood driven delivery and non-specific delivery of IgG control. Using the IS index calculation: (%ID/g VCAM-1-organ/%ID/g VCAM-1-blood)/( (%ID/g IgG-organ/%ID/g IgG-blood), we found that in sham animals, the IS index of brain and spleen are similar (2.539±0.431 vs. 2.251±0.082, p=0.055). However, in TBI animals, the IS index of brain in significantly higher than spleen (14.805±1.273 vs. 9.735±0.657, p=0.024). It suggests that VCAM-1-targeted liposomes have specific targeting to the TBI-injured brain.

1. The introduction needs more clarity regarding what the authors are targeting. For example, is the goal to just target inflamed endothelium in the BBB or cross the BBB. Seems like the focus is targeting endothelium. If that’s true, what is the reason for discussing nanocarriers developed previously for BBB permeation.

We thank the reviewer for the comment, and we agree that our focus is to target endothelium. To avoid confusion, we rewrote the introduction to minimize the discussion of BBB-crossing nanocarriers.

2. For data in Fig. 1D and E, authors should show representative images of brain to show antibody accumulation?

We appreciate the reviewer for the comment on representative images of antibody accumulation. In this study, instead of using fluorescent tracing, we used isotope tracing (^125^I), which allows us to quantitatively measure the tissue uptake without extra procedures of tissue homogenization. At this point, we consider the representative images will not provide additional information, and thus could not ethically justify the use of extra animals. However, we agree that spatial location of antibody and nanocarrier in the context of TBI is very important for the translational study, and will included in the future study.

3. The authors mention that “VCAM antibody were taken up ~2.5 fold more than IgG. These lower fold improvements in TBI were driven by an increased IgG accumulation in the brain, which is likely due to vascular leak of plasma into brain parenchyma”. This seems more of speculation. Would be great to add imaging data to prove this.

As discussed in Comment 1 from Reviewer 1 above: In order to demonstrate such disruption, we compared the accumulation of untargeted IgG antibody in naïve vs. TBI brain. IgG has a half-life of 6-8 days in the blood stream, and our data shows that only a tiny fraction enters the brain in naïve mice. However, when there is capillary leakage, IgG will extravasate into the brain together with other plasma protein. The result below shows ~8-fold increase of IgG in the TBI brain, when compared to the naïve brain, suggesting the disruption of BBB. Dashed lines representing the naïve level for IgG and anti-VCAM-1 antibody have been added into Fig 1D and 1E, and the following text has been added in the Results section.

It is worth noting that the IgG accumulation in the TBI brain is ~8 fold higher than that of naïve brain (black dashed line). Notably, IgG circulates well and accumulates minimally in the healthy brain. These results suggested that TBI induces capillary leakage, though at this point we cannot say if IgG extravasation into the TBI brain is due to transport that is transcellular, paracellular, or via frank hemorrhage. Additionally, the extravasated IgG in TBI brain contributed to the lower fold-improvements of VCAM-1 antibody vs. IgG control in TBI vs. sham brain.

4. The authors say “liposomes, which are FDA approved particles”. Particles are not approved by the FDA, drugs are. It would be better to say “which are present in FDA approved formulations”

It has been corrected in the main text.

5. The authors describe results in Fig. 2A as tissue/brain uptake and also mention
“brain accumulation”, which are not technically correct as their imaging data shows particles binding to endothelium.

We thank the reviewer for pointing this out. We used the term brain uptake/accumulation, because of its anatomical localization. Brain endothelium is part of brain tissue. We never claimed that we are reaching the brain parenchyma. In fact, our approach is to target the brain vasculature because it is easily accessible after an intravascular administration.

6. Data for Vcam mAb in 2C seems like the exact same data in 1D and E. Similarly the data for Vcam liposome in 2C is exactly same as 2A. There is no need for duplication of data

Figure 2C has been replaced with the calculation of immunospecificity index. This calculation (see the equation below) is able to eliminate the effect of blood driven delivery and non-specific delivery of IgG control, and provide us better understanding of targeting specificity of VCAM-1.

Immunospecificity: ${\frac{\text{\%ID/g VCAM-organ}}{\text{\%ID/g VCAM-blood}}}/{\frac{\text{\%ID/g IgG-organ}}{\text{\%ID/g IgG-blood}}}$

7. The difference between 2E and F is not clear.

The following text has been added into the Results section to clarify the difference of Fig 2E and 2F:

In terms of cellular distribution of VCAM-1-liposomes (**Fig 2E**), out of 100% liposome-positive cells, 53.55 ± 1.255% were endothelial cells, 41.24 ± 1.96% were leukocytes and 1.94 ± 0.67% were microglia. In terms of cell uptake specificity (**Fig 2F**), within the certain cell type, 19.07 ± 2.06% of endothelial cells took up VCAM-1-liposomes compared to only 3.33 ± 0.08% of leukocytes and 0.05 ± 0.17% of microglia.
