# Supplementary material for: Targeting of nanoparticles to the cerebral vasculature after traumatic brain injury
Source: PLoS One. 2024 Jun 10;19(6):e0297451. doi: 10.1371/journal.pone.0297451 (PMC11164327; doi:10.1371/journal.pone.0297451)
Supplement: Attachment — Submitted filename: Response2 to reviewer final.docx [file pone.0297451.s006.docx]

**Second response to reviewer’s comments**

We thank the reviewers their positive feedback. Below we address the remaining points that were raised.

Reviewer #1: I think the authors revised the ms thoroughly and generally replied adequately to the criticism made by the reviewers

Reviewer #2: In this revised manuscript, most points were improved. However, some minor points are needed to be revised for the publication.

1) In the Table S2, value of the brain uptake for VCAM liposome (sham) may be not consistent with that of Figure 2A.
Please check carefully.

Thank you for picking up this error. We have corrected Table S2.

2) Figure Legend: In the Fig 1(F), “PECAM” is described although data is not shown in the text and Figures. Please delete if data of PECAM is not shown.

We have deleted PECAM in the figure legend.

3) In the Fig 2C, I wonder why targeting specificity of VCAM mAb was deceased by TBI.

Great point. The targeting specificity is calculated as (%ID/g VCAM-1-organ/%ID/g VCAM-1-blood)/(%ID/g IgG-organ/%ID/g IgG-blood). Due to the leaky blood-brain barrier after TBI, IgG uptake in the TBI brain is 1.86-fold higher than in sham brain. When antibody uptake in other tissues are comparable (e.g. the blood %ID/g of IgG and VCAM-1 antibody are within the range of what we’ve been observed in mice), the small change in brain uptake will significantly impact on the calculated outcome of the immunospecificity index. We have added this explanation into the Results section.

Reviewer #3: (No Response)
