# Supplementary material for: Targeting of nanoparticles to the cerebral vasculature after traumatic brain injury
Source: PLoS One. 2024 Jun 10;19(6):e0297451. doi: 10.1371/journal.pone.0297451 (PMC11164327; doi:10.1371/journal.pone.0297451)
Supplement: Attachment — Submitted filename: Response to editor.docx [file pone.0297451.s007.docx]

**Response to editor’s comments**

I have a request regarding the reproduction of the images in Figure 1 from another article. You state that the images are reproduced with permission [39, 40].
You should indicate which images are taken from existing articles. Also indicate that you have permission to reproduce the images from both the authors and the publishers of the articles from which the images are taken.
Please obtain written permission from both the authors and the publishers of the articles from which the images are taken. Please include the written permissions with your submission.

We thank the editor for the feedback and guidance regarding the reproduction of images in Figure 1. We appreciate your thorough review, and we have revised the manuscript and confirmed that all the images in the updated Figure 1 (see below) are original and not taken from existing articles. We have removed the incorrect statement in the caption of Figure 1.

Please confirm at this time whether or not your submission contains all raw data required to replicate the results of your study. Authors must share the “minimal data set” for their submission. PLOS defines the minimal data set to consist of the data required to replicate all study findings reported in the article, as well as related metadata and methods (<https://journals.plos.org/plosone/s/data-availability#loc-minimal-data-set-definition>).

We thank the editor for pointing it out. We have changed the figures with individual dots to present all the raw data included in this manuscript in both main figures and supporting document.
